# Supplementary material for: Stable hydrogen isotopes record the summering grounds of eastern red bats (Lasiurus borealis)
Source: PeerJ. 2014 Oct 16;2:e629. doi: 10.7717/peerj.629 (PMC4203026; doi:10.7717/peerj.629)
Supplement: Table S1 [file peerj-02-629-s001.docx]

| **Catalog Number** | **Date Collected** | **Sex** | **State** | **Decimal Latitude** | **Decimal Longitude** | **Elevation (m)** | **δ^2^H Growing Season** | **δ^2^H Hair** | **Included in June 14-August 7 RMA** |
| --- | --- | --- | --- | --- | --- | --- | --- | --- | --- |
|  |  |  |  |  |  |  |  |  |  |
| 202189 | 6/4/1913 | Female | Alabama | 33.458 | -87.357 | 57 | -16.33 | 6.59 |  |
| 176511 | 6/9/1911 | Male | Alabama | 31.572 | -85.25 | 138 | -18.67 | -60.60 |  |
| 171424 | 6/9/1911 | Female | Alabama | 31.572 | -85.25 | 138 | -18.67 | -11.71 |  |
| 201598 | 6/12/1913 | Female | Alabama | 34.022 | -86.089 | 167 | -16.33 | -18.63 |  |
| 171491 | 6/17/1911 | Male | Alabama | 33.017 | -86.312 | 199 | -17.67 | -2.70 | Y |
| 201709 | 6/17/1913 | Female | Alabama | 34.358 | -86.295 | 196 | -16.33 | -6.97 | Y |
| 201710 | 6/22/1913 | Female | Alabama | 34.138 | -87.006 | 224 | -17.33 | -19.50 | Y |
| 208138 | 7/31/1915 | Female | Alabama | 32.184 | -86.58 | 74 | -17.67 | -14.50 | Y |
| 203166 | 8/1/1913 | Unknown | Alabama | 30.694 | -88.043 | 2 | -20.00 | -9.15 |  |
| 208139 | 8/3/1915 | Female | Alabama | 32.184 | -86.58 | 74 | -17.67 | -7.27 | Y |
| 505297 | 7/5/1972 | Female | District of Columbia | 38.907 | -77.036 | 19 | -26.67 | -33.63 | Y |
| 216644 | 7/11/1916 | Male | District of Columbia | 38.907 | -77.036 | 19 | -26.67 | -34.48 | Y |
| 175508 | 8/15/1912 | Female | District of Columbia | 38.907 | -77.036 | 19 | -26.67 | -33.83 |  |
| 314874 | 8/18/1960 | Male | District of Columbia | 38.898 | -77.018 | 12 | -26.67 | -38.02 |  |
| 314875 | 8/25/1960 | Male | District of Columbia | 38.898 | -77.018 | 12 | -26.67 | -47.83 |  |
| 276648 | 8/28/1944 | Female | District of Columbia | 38.907 | -77.036 | 19 | -26.67 | -38.03 |  |
| 248960 | 7/11/1929 | Unknown | Florida | 30.421 | -87.217 | 18 | -20.33 | -25.37 |  |
| 268953 | 7/14/1939 | Male | Florida | 29.217 | -82.058 | 14 | -22.33 | -24.86 | Y |
| 208140 | 8/9/1915 | Female | Florida | 30.6 | -87.4 | 6 | -20.33 | -4.12 |  |
| 247332 | 6/25/1927 | Female | Georgia | 34.766 | -84.77 | 227 | -16.67 | -24.34 | Y |
| 254344 | 7/15/1901 | Female | Georgia | 33.707 | -84.435 | 317 | -17.00 | -18.30 | Y |
| 254345 | 8/6/1901 | Male | Georgia | 33.707 | -84.435 | 317 | -17.00 | -14.59 | Y |
| 254346 | 8/13/1901 | Female | Georgia | 33.707 | -84.435 | 317 | -17.00 | 3.90 |  |
| 114790 | 7/23/1901 | Female | Illinois | 40.9 | -91.05 | 162 | -33.33 | -29.85 | Y |
| 114792 | 7/25/1901 | Male | Illinois | 40.9 | -91.05 | 162 | -33.33 | -32.91 | Y |
| 114791 | 7/25/1901 | Male | Illinois | 40.9 | -91.05 | 162 | -33.33 | -31.77 | Y |
| 363838 | 6/24/1961 | Male | Indiana | 39.524 | -87.125 | 200 | -26.33 | -32.20 | Y |
| 363714 | 6/28/1962 | Female | Indiana | 41.55 | -87.51 | 187 | -31.67 | -48.64 | Y |
| 363717 | 7/10/1962 | Female | Indiana | 40.45 | -85.445 | 276 | -30.33 | -49.54 | Y |
| 363716 | 7/10/1962 | Female | Indiana | 40.45 | -85.445 | 276 | -30.33 | -29.29 | Y |
| 363836 | 7/12/1960 | Female | Indiana | 40.417 | -86.875 | 213 | -29.33 | -57.62 | Y |
| 363846 | 7/16/1962 | Female | Indiana | 38.252 | -87.219 | 172 | -23.00 | -34.90 | Y |
| 363848 | 7/19/1962 | Female | Indiana | 40.422 | -86.264 | 257 | -29.67 | -44.85 | Y |
| 363734 | 7/25/1962 | Female | Indiana | 39.774 | -86.140 | 219 | -27.67 | -37.14 | Y |
| 363719 | 7/26/1962 | Male | Indiana | 41.359 | -85.666 | 265 | -32.33 | -33.77 | Y |
| 363839 | 7/27/1961 | Female | Indiana | 40.104 | -87.084 | 226 | -28.33 | -41.07 | Y |
| 363726 | 8/2/1962 | Male | Indiana | 41.16 | -86.61 | 218 | -31.00 | -33.56 | Y |
| 363843 | 8/9/1961 | Male | Indiana | 41.181 | -86.924 | 215 | -31.00 | -32.09 |  |
| 363727 | 8/13/1962 | Female | Indiana | 38.945 | -84.989 | 257 | -26.00 | -45.86 |  |
| 363732 | 8/15/1962 | Female | Indiana | 38.905 | -84.987 | 268 | -26.00 | -37.16 |  |
| 363849 | 8/24/1962 | Male | Indiana | 39.249 | -86.742 | 208 | -26.00 | -36.76 |  |
| 297189 | 7/24/1907 | Male | Kansas | 38.971 | -95.23 | 251 | -36.67 | -39.52 | Y |
| 297190 | 7/30/1907 | Male | Kansas | 38.971 | -95.23 | 251 | -36.67 | -37.63 | Y |
| 297191 | 8/2/1907 | Male | Kansas | 38.971 | -95.23 | 251 | -36.67 | -39.67 | Y |
| 169454 | 7/29/1910 | Female | Kentucky | 38.405 | -82.6 | 169 | -25.00 | -33.45 | Y |
| 223145 | 6/16/1916 | Female | Maryland | 38.969 | -77.142 | 43 | -27.33 | -32.01 | Y |
| 204979 | 7/15/1914 | Female | Maryland | 38.97 | -77.177 | 24 | -27.00 | -30.75 | Y |
| 127860 | 7/19/1903 | Male | Maryland | 38.974 | -76.495 | 7 | -26.67 | -39.66 | Y |
| 560460 | 7/26/1968 | Female | Maryland | 38.291 | -76.638 | 27 | -25.33 | -39.36 | Y |
| 568880 | 8/14/2003 | Female | Maryland | 38.97 | -77.184 | 22 | -27.00 | 8.00 |  |
| 160616 | 8/15/1909 | Male | Maryland | 38.97 | -77.177 | 24 | -27.00 | -27.57 |  |
| 122185 | 8/21/1903 | Male | Maryland | 38.97 | -77.177 | 24 | -27.00 | 2.27 |  |
| 551794 | 8/10/1980 | Male | Michigan | 46.278 | -86.072 | 218 | -44.67 | -42.50 |  |
| 551795 | 8/10/1980 | Female | Michigan | 46.278 | -86.072 | 218 | -44.67 | -38.86 |  |
| 551796 | 8/10/1980 | Female | Michigan | 46.278 | -86.072 | 218 | -44.67 | -38.21 |  |
| 234025 | 8/13/1919 | Male | Minnesota | 46.876 | -96.764 | 275 | -60.67 | -24.42 |  |
| 234026 | 8/21/1919 | Male | Minnesota | 46.876 | -96.764 | 275 | -60.67 | -28.02 |  |
| 347545 | 6/9/1970 | Female | Mississippi | 31.87 | -89.995 | 81 | -20.67 | -22.73 |  |
| 347546 | 6/9/1970 | Female | Mississippi | 31.87 | -89.995 | 81 | -20.67 | -21.04 |  |
| 347547 | 6/9/1970 | Female | Mississippi | 31.87 | -89.995 | 81 | -20.67 | -20.07 |  |
| 349271 | 6/22/1972 | Female | Mississippi | 33.45 | -88.818 | 103 | -18.33 | -41.45 | Y |
| 348356 | 6/30/1972 | Female | Mississippi | 31.1 | -91.507 | 65 | -22.33 | -21.84 | Y |
| 348357 | 6/30/1972 | Female | Mississippi | 31.1 | -91.507 | 65 | -22.33 | -21.35 | Y |
| 348360 | 7/2/1972 | Female | Mississippi | 31.1 | -91.507 | 65 | -22.33 | -31.84 | Y |
| 348363 | 7/2/1972 | Female | Mississippi | 31.1 | -91.507 | 65 | -22.33 | -28.38 | Y |
| 348364 | 7/2/1972 | Female | Mississippi | 31.1 | -91.507 | 65 | -22.33 | -24.06 | Y |
| 348358 | 7/2/1972 | Female | Mississippi | 31.1 | -91.507 | 65 | -22.33 | -22.09 | Y |
| 348366 | 7/2/1972 | Female | Mississippi | 31.1 | -91.507 | 65 | -22.33 | -21.85 | Y |
| 348362 | 7/2/1972 | Female | Mississippi | 31.1 | -91.507 | 65 | -22.33 | -19.46 | Y |
| 348361 | 7/2/1972 | Female | Mississippi | 31.1 | -91.507 | 65 | -22.33 | -19.42 | Y |
| 348359 | 7/2/1972 | Female | Mississippi | 31.1 | -91.507 | 65 | -22.33 | -18.89 | Y |
| 348365 | 7/2/1972 | Female | Mississippi | 31.1 | -91.507 | 65 | -22.33 | -18.28 | Y |
| 180156 | 8/9/1912 | Male | Mississippi | 31.373 | -90.24 | 116 | -21.67 | -30.53 |  |
| 554360 | 6/4/1968 | Female | Missouri | 40.289 | -94.787 | 335 | -38.67 | -35.61 |  |
| 111070 | 7/14/1900 | Female | New York | 42.967 | -75.688 | 396 | -41.67 | -38.88 | Y |
| 111071 | 7/31/1900 | Female | New York | 42.967 | -75.688 | 396 | -41.67 | -63.02 | Y |
| 140763 | 8/16/1900 | Female | New York | 42.967 | -75.688 | 396 | -41.67 | -58.55 |  |
| 254343 | 8/22/1900 | Female | New York | 41.392 | -73.956 | 47 | -34.00 | -41.47 |  |
| 140764 | 8/30/1900 | Female | New York | 42.967 | -75.688 | 396 | -41.67 | -33.78 |  |
| 212750 | 6/26/1916 | Male | North Carolina | 35.246 | -81.253 | 238 | -19.67 | -16.30 | Y |
| 266473 | 8/9/1939 | Male | North Dakota | 48.582 | -100.728 | 441 | -75.33 | -34.79 |  |
| 273724 | 6/14/1938 | Female | Oklahoma | 34.708 | -98.677 | 472 | -40.00 | -32.49 | Y |
| 133140 | 6/21/1904 | Female | Oklahoma | 36.099 | -96.179 | 256 | -34.67 | -24.15 | Y |
| 133141 | 6/22/1904 | Female | Oklahoma | 36.099 | -96.179 | 256 | -34.67 | -35.26 | Y |
| 133142 | 6/23/1904 | Female | Oklahoma | 36.099 | -96.179 | 256 | -34.67 | -28.95 | Y |
| 133144 | 6/29/1904 | Unknown | Oklahoma | 36.099 | -96.179 | 256 | -34.67 | -40.78 |  |
| 100683 | 6/20/1900 | Female | Pennsylvania | 40.695 | -80.305 | 242 | -33.67 | -29.31 | Y |
| 282670 | 7/17/1947 | Male | Pennsylvania | 41.087 | -75.328 | 366 | -36.33 | -28.26 | Y |
| 282671 | 7/26/1947 | Male | Pennsylvania | 40.291 | -77.11 | 176 | -32.67 | -35.69 | Y |
| 212747 | 6/14/1916 | Male | South Carolina | 34.92 | -82.603 | 343 | -19.00 | -11.01 | Y |
| 159049 | 8/18/1908 | Male | Tennessee | 36.178 | -84.185 | 279 | -19.00 | -37.65 |  |
| 117376 | 6/11/1902 | Male | Texas | 32.757 | -94.345 | 59 | -25.00 | -13.12 |  |
| 117375 | 6/12/1902 | Male | Texas | 32.757 | -94.345 | 59 | -25.00 | -6.90 |  |
| 117411 | 6/24/1902 | Female | Texas | 29.893 | -99.22 | 613 | -32.67 | -18.62 | Y |
| 117413 | 7/3/1902 | Female | Texas | 30.077 | -99.24 | 526 | -31.67 | -18.25 | Y |
| 117412 | 7/3/1902 | Female | Texas | 30.077 | -99.24 | 526 | -31.67 | -10.68 | Y |
| 169711 | 7/6/1910 | Unknown | Texas | 32.78 | -96.8 | 130 | -27.67 | -26.18 |  |
| 570886 | 8/25/2009 | Female | Texas | 32.897 | -97.041 | 177 | -28.67 | 0.66 |  |
| 293376 | 8/3/1939 | Male | Virginia | 36.641 | -80.266 | 406 | -25.00 | -22.94 | Y |
| 293377 | 8/5/1939 | Male | Virginia | 36.79 | -80.277 | 432 | -25.33 | -23.64 | Y |
| 293375 | 8/8/1944 | Male | Virginia | 37.23 | -80.414 | 634 | -28.33 | -35.63 |  |
| 258995 | 8/24/1934 | Male | Virginia | 38.896 | -77.118 | 102 | -27.67 | -16.72 |  |
| 297853 | 8/26/1951 | Male | Virginia | 38.608 | -78.358 | 784 | -34.33 | -18.99 |  |
| 347653 | 8/13/1971 | Female | West Virginia | 38.745 | -78.968 | 647 | -33.33 | -45.91 |  |
| 347654 | 8/13/1971 | Female | West Virginia | 38.745 | -78.968 | 647 | -33.33 | -38.68 |  |
| 347663 | 8/14/1971 | Female | West Virginia | 38.745 | -78.968 | 647 | -33.33 | -35.74 |  |
| 347662 | 8/14/1971 | Male | West Virginia | 38.745 | -78.968 | 647 | -33.33 | -30.57 |  |
| 347661 | 8/14/1971 | Male | West Virginia | 38.745 | -78.968 | 647 | -33.33 | -29.86 |  |
| 347660 | 8/14/1971 | Male | West Virginia | 38.745 | -78.968 | 647 | -33.33 | -24.16 |  |
| 234847 | 8/3/1920 | Male | Wisconsin | 42.59 | -90.563 | 293 | -39.00 | -36.05 | Y |
| 234848 | 8/4/1920 | Female | Wisconsin | 42.59 | -90.563 | 293 | -39.00 | -35.19 | Y |
| 147250 | 8/5/1901 | Female | Wisconsin | 42.633 | -88.644 | 285 | -37.67 | -46.53 | Y |
